# MRI Response Assessment in Glioblastoma Patients Treated with Dendritic-Cell-Based Immunotherapy

**DOI:** 10.3390/cancers14061579

**Published:** 2022-03-20

**Authors:** Johanna Heugenhauser, Malik Galijasevic, Stephanie Mangesius, Georg Goebel, Johanna Buchroithner, Friedrich Erhart, Josef Pichler, Georg Widhalm, Günther Stockhammer, Sarah Iglseder, Christian F. Freyschlag, Stefan Oberndorfer, Karin Bordihn, Gord von Campe, Thomas Czech, Birgit Surböck, Tadeja Urbanic Purkart, Christine Marosi, Thomas Felzmann, Martha Nowosielski

**Affiliations:** 1 Department of Neurology, Medical University of Innsbruck, 6020 Innsbruck, Austria; johanna.heugenhauser@i-med.ac.at (J.H.); guenther.stockhammer@i-med.ac.at (G.S.); sarah.iglseder@i-med.ac.at (S.I.); 2 Department of Neuroradiology, Medical University of Innsbruck, 6020 Innsbruck, Austria; malik.galijasevic@i-med.ac.at; 3 Neuroimaging Core Facility, Medical University of Innsbruck, 6020 Innsbruck, Austria; 4 Department of Medical Statistics, Informatics and Health Economics, Medical University of Innsbruck, 6020 Innsbruck, Austria; georg.goebel@i-med.ac.at; 5 Department of Neurosurgery, Johannes Kepler University, 4020 Linz, Austria; johanna.buchroithner@kepleruniklinikum.at; 6 Department of Neurosurgery, Medical University of Vienna, 1090 Vienna, Austria; friedrich.erhart@meduniwien.ac.at (F.E.); georg.widhalm@meduniwien.ac.at (G.W.); thomas.czech@meduniwien.ac.at (T.C.); 7 Department of Neurology, Medical University of Vienna, 1090 Vienna, Austria; 8 Department of Internal Medicine and Neurooncology, Johannes Kepler University, 4020 Linz, Austria; josef.pichler@kepleruniklinikum.at; 9 Department of Neurosurgery, Medical University of Innsbruck, 6020 Innsbruck, Austria; christian.freyschlag@i-med.ac.at; 10 Department of Neurology, Landsteiner Private University, 3100 St. Pölten, Austria; stefan.oberndorfer@stpoelten.lknoe.at; 11 Department of Neurosurgery, Paracelsus Private Medical University, 5020 Salzburg, Austria; k.bordihn@salk.at; 12 Department of Neurosurgery, Medical University of Graz, 8036 Graz, Austria; gord.von-campe@medunigraz.at; 13 Department of Neurology, Klinik Favoriten, Wiener Gesundheitsverbund, 1100 Vienna, Austria; birgit.surboeck@gesundheitsverbund.at; 14 Department of General Neurology and Neuroradiology, Vascular and Interventional Radiology, Medical University of Graz, 8036 Graz, Austria; tadeja.urbanic-purkart@medunigraz.at; 15 Clinical Division of Medical Oncology, Department for Internal Medicine I, Medical University of Vienna, 1090 Vienna, Austria; christine.marosi@meduniwien.ac.at; 16 Activartis Biotech GmbH, 1160 Vienna, Austria; office@activartis.com

**Keywords:** radiologic response criteria, immunotherapy, glioblastoma, iRANO, mRANO, volumetric measurements

## Abstract

**Simple Summary:**

In order to compare responses to different therapies among clinical trials and to differentiate between therapy-induced changes and true tumor progression, reliable response parameters are crucial. With the advent of targeted and immunologic treatments, several assessment tools have been proposed. In this post hoc analysis we compared assessment criteria according to MacDonald, RANO, mRANO, iRANO as well as Vol-RANO and Vol-mRANO in patients with newly diagnosed glioblastoma treated with standard of care (SOC) ± tumor lysate-charged autologous dendritic cells (Audencel). We found that the best correlation between progression-free survival (PFS) and overall survival (OS) was seen for mRANO and Vol-mRANO. Interestingly, iRANO was not superior for predicting OS in patients treated with Audencel.

**Abstract:**

Introduction: In this post hoc analysis we compared various response-assessment criteria in newly diagnosed glioblastoma (GB) patients treated with tumor lysate-charged autologous dendritic cells (Audencel) and determined the differences in prediction of progression-free survival (PFS) and overall survival (OS). Methods: 76 patients enrolled in a multicenter phase II trial receiving standard of care (SOC, *n* = 40) or SOC + Audencel vaccine (*n* = 36) were included. MRI scans were evaluated using MacDonald, RANO, Vol-RANO, mRANO, Vol-mRANO and iRANO criteria. Tumor volumes (T1 contrast-enhancing as well as T2/FLAIR volumes) were calculated by semiautomatic segmentation. The Kruskal-Wallis-test was used to detect differences in PFS among the assessment criteria; for correlation analysis the Spearman test was used. Results: There was a significant difference in median PFS between mRANO (8.6 months) and Vol-mRANO (8.6 months) compared to MacDonald (4.0 months), RANO (4.2 months) and Vol-RANO (5.4 months). For the vaccination arm, median PFS by iRANO was 6.2 months. There was no difference in PFS between SOC and SOC + Audencel. The best correlation between PFS/OS was detected for mRANO (r = 0.65) and Vol-mRANO (r = 0.69, each *p* < 0.001). A total of 16/76 patients developed a pure T2/FLAIR progressing disease, and 4/36 patients treated with Audencel developed pseudoprogression. Conclusion: When comparing different response-assessment criteria in GB patients treated with dendritic cell-based immunotherapy, the best correlation between PFS and OS was observed for mRANO and Vol-mRANO. Interestingly, iRANO was not superior for predicting OS in patients treated with Audencel.

## 1. Introduction

Glioblastoma (GB) is the most frequent primary brain tumor in adults [1,2]. Despite multimodal treatment, life expectancy is still poor [3,4,5]. Considering the enormous progress in cancer immunotherapy during the past few years, a number of new immunologic treatment approaches, including personalized cell vaccines, are currently under investigation for GB. Unfortunately, no significant improvement in overall survival (OS) or progression-free survival (PFS) has been observed so far [6,7,8,9,10,11,12,13,14]. To compare the treatment responses between different therapies among clinical trials and to differentiate between therapy-induced changes and true tumor progression, reliable response parameters are crucial. Magnetic resonance imaging (MRI) is the gold standard for evaluating response and progression during treatment. However, different treatments, in particular radiotherapy combined with temozolomide chemotherapy as well as immunologic strategies, challenge the current imaging response criteria. Pseudoprogression (PsP), a subacute treatment-related phenomenon, results from a disruption of the blood–brain barrier and presents an increased contrast enhancement on MRI, mimicking tumor progression [15]. PsP was reported in up to 10–30% of GB patients following radiochemotherapy [16,17]. Other than that, patients treated with antiangiogenic therapies often show a decrease in contrast enhancement but without a true tumor response, also referred to as a pseudoresponse (PrP). Frequently, progression is only observable as a non-enhancing abnormality in T2-weighted or fluid-attenuated inversion recovery (FLAIR) image sequences in those patients [18].

In recent years, several radiologic assessment tools have been proposed [19]. In 1990 the MacDonald criteria were introduced, using two-dimensional tumor measurements, as well as corticosteroid use and the clinical performance of the patient for response assessment [20]. Twenty years later, the Response Assessment in Neuro-Oncology (RANO) criteria were proposed [21], utilizing T2-weighted or FLAIR image sequences to account for non-enhancing tumor components and therapy-induced MRI changes such as PsP and PrP [21,22]. To better account for the phenomenon of PsP, the modified RANO (mRANO) criteria were proposed in 2017, which require a confirmation scan to better capture the occurrence of true tumor progression or PsP in GB patients [23]. With the advent of immunotherapies, unique patterns of responses were observed during the treatment of systemic cancer. Especially within the first weeks after starting immunotherapy the appearance of new local or distant lesions or an increase in existing lesions may simply reflect an immune-mediated phenomenon rather than true tumor progression [24]. In consideration of such PsP during immunotherapy of GB, the Immunotherapy RANO (iRANO) criteria [25] were developed. Interestingly, the iRANO criteria were developed before the true incidence of PsP during immunotherapy was established, which in consecutive studies was found to range between 10–15% [26,27]. So far, only a few studies [28,29] exist, which directly compare and evaluate currently available response criteria.

In order to identify the best response assessment for GB patients treated by immunotherapy in addition to standard of care, we performed a post hoc MRI analysis of a multicenter phase II clinical trial of newly diagnosed GB patients treated with tumor lysate-charged autologous dendritic cells (Audencel) [14]. Under the hypothesis to detect differences in prediction of PFS and OS, we compared the imaging data sets according to the various response-assessment criteria currently in clinical use (MacDonald, RANO, Vol-RANO, mRANO, Vol-mRANO, iRANO). In addition, we evaluated the number of patients showing a pure T2/FLAIR progressing disease as well as the number of patients with PsP using these different response-assessment tools.

## 2. Materials and Methods

### 2.1. Patients

We retrospectively analyzed imaging data from patients with newly diagnosed GB WHO 4. All patients were enrolled in a national randomized multicenter open-label phase II glioblastoma dendritic-cell vaccine (Audencel) trial [14] and were treated with either standard of care (SOC) or SOC + Audencel dendritic-cell (DC) vaccine. SOC treatment included maximal safe surgical resection followed by radiochemotherapy according to the “Stupp protocol” [3]. The clinical data of the trial as well as the manufacturing of the dendritic cells were published previously [14]. In summary, the Audencel DC vaccine is a personalized cell-based immunotherapy, using pulsed dendritic cells cultivated from peripheral blood mononuclear cells from each individual patient and co-incubated with autologous tumor lysates, harbored from surgically resected tumor samples. Further, Audencel DC vaccine therapy was started in week seven with three boosting doses weekly followed by a monthly interval. All patients gave written informed consent before trial entry and were observed until death or for at least 12 months. The study was reviewed and approved by the local independent ethics committee and institutional review board.

### 2.2. Magnetic Resonance Imaging

Owing to the multicenter design of the trial, multiple MRI systems were used, all operating at a 1.5T field strength. A 3D T1-weighted imaging protocol including magnetization-prepared rapid acquisition with gradient echo (MP-RAGE) MRI sequences or T1-weighted spin-echo (SE) images with contrast enhancement (0.1 mmol/kg gadopentetate dimeglumine) was performed at each center, resulting in images of similar resolution, with a slice thickness of at least 1.5 mm. All patients underwent axial T2- or FLAIR-weighted imaging with a slice thickness of at least 2 mm. Baseline MRI was carried out before surgery and within 48 h after surgery. The first follow-up imaging was carried out 10 weeks ± two weeks after the start of radiochemotherapy, followed by a neuroradiological examination every three months ± two weeks. In this post hoc analysis, imaging data was analyzed until death/loss to follow-up of each patient, or until the end of trial in June 2015.

### 2.3. Radiologic Response Assessment

**Figure 1:**
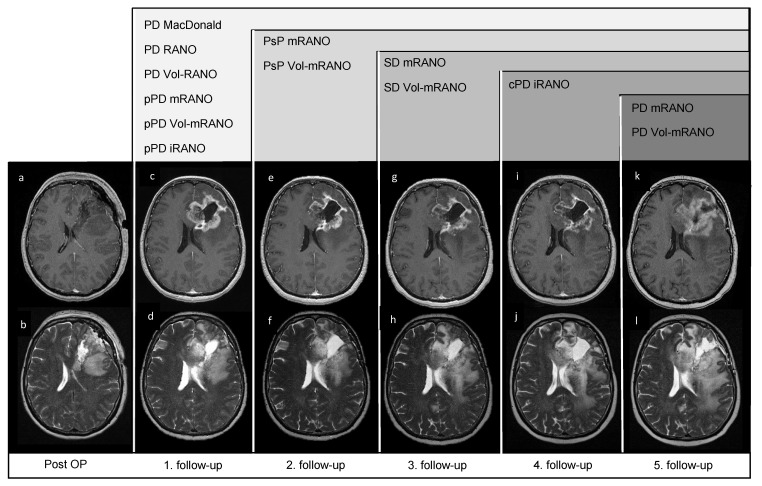
Post-OP and follow-up MRI scans of patient VAX_0083 (Audencel-arm): T2- (**b**,**d**,**f**,**h**,**j**,**l**) and postgadolinium T1-weighted MRI sequences (**a**,**c**,**e**,**g**,**i**,**k**) are displayed. This figure illustrates the different time points of progression by different response assessments. At the first follow-up scan (**c**,**d**), a new contrast-enhancing lesion is seen on postgadolinium T1-weighted MRI sequences (**c**) and a significant increase or ≥100% increase in volume of non-enhancing abnormalities (**d**) compared to the post-OP scan (baseline, (**b**)) is seen. Progressive disease (PD) by MacDonald, RANO, Vol-RANO, while preliminary progressive disease (pPD) by mRANO, Vol-mRANO is diagnosed. Because the first follow-up MRI (**c**,**d**) is within the first six weeks of immunotherapy-treatment start, pPD by iRANO is defined. In the second follow-up MRI (**e**,**f**) this patient is diagnosed with pseudoprogression (PsP) because no further ≥25% increase in the cross-section area or ≥40% increase in total volume of the contrast-enhancing tumor mass is seen (**e**) compared to the first follow-up MRI (**c**). In the third follow-up MRI (**g**,**h**) the contrast-enhancing tumor mass does not increase in size (**g**) compared to the second follow-up MRI (**e**), hence stable disease (SD) by mRANO and Vol-mRANO is defined. In the fourth follow-up MRI (**i**,**j**), confirmed progressive disease (cPD) by iRANO is defined, as a significant increase in non-enhancing abnormalities (**j**) compared to the post-OP scan (baseline, (**b**)) is seen and this scan (**i**,**j**) is ≥3 months after the first follow-up MRI (**c**,**d**), where pPD by iRANO was diagnosed. In the fifth follow-up MRI (**k**,**l**) a ≥25% increase in the cross-section area or ≥40% increase in total volume of the contrast-enhancing tumor mass (**k**) compared to the second follow-up MRI (**e**) is seen and PD by mRANO and Vol-mRANO is diagnosed.

Response assessment was performed by two neuroradiologists (M.G., S.M.), who were blinded to the patients’ clinical information and outcome and reviewed all MRI scans independently from each other. In case of disagreement during consensus reading, expert opinion from a third neuroradiologist was obtained. For radiologic response assessment, two-dimensional (2D) and recently also three-dimensional (3D) assessment criteria are suggested in guidelines [20,21,23,25]. In this analysis we also included 3D assessment criteria to evaluate whether these methods are more precise in predicting OS compared to the more widely used 2D criteria. For two-dimensional (2D) response-assessment criteria MacDonald [20], RANO [21], mRANO [23] and iRANO [25], and for three-dimensional (3D) assessment methods Vol-mRANO [23] and Vol-RANO [30], were applied to each available MRI scan obtained from each patient (postoperative and follow-up MRI). For each of the 2D response-assessment criteria, axial slices of postgadolinium isovoxel T1-weighted MRI scans were used to evaluate the cross-section area of the contrast-enhancing tumor mass. The cross-section area was calculated as the product of the largest measurable diameter and the perpendicular diameter of the enhancing tumor in the same axial slice [20,21,23,25]. Measurable disease was defined as both diameters ≥1 cm [21]. Additionally, for RANO and iRANO criteria, axial slices of T2/FLAIR MRI sequences were analyzed [21,25]. A summary of the different assessment criteria is displayed in Table 1. Each patient was classified as either complete response (CR), partial response (PR), stable disease (SD) or progressive disease (PD) according to the six different assessment criteria (MacDonald, RANO, Vol-RANO, mRANO, Vol-mRANO, iRANO) at every available follow-up MRI scan. Figure 1 illustrates the different time points of progression by different response-assessment criteria in a representative patient (VAX_0083, Audencel-arm). The date of the first follow-up scan showing PD (confirmed PD for mRANO and Vol-mRANO) was used as date of progression. If the patient showed SD at the last scan obtained, the patient was censored by using the date of the last scan as date of progression. If a re-resection was carried out, the last scan prior to re-resection was used as the date of PD if the patient was not diagnosed with PD earlier. PFS was calculated from the date of pre-surgery MRI (date of diagnosis) to the date of disease progression, and OS was calculated from the date of pre-surgery MRI (date of diagnosis) to time of death/loss to follow-up. Ten patients who were lost for follow-up were censored using the date of last visit as time of death. Two patients who were still alive at the end of the trial were censored by using the date of study closure (1 June 2015).

### 2.4. Volumetric Measurement

For 3D response assessment, total contrast-enhancing tumor volume (excluding dura, blood vessels, the resection cavity and/or central necrotic area) was calculated on postgadolinium isovoxel T1-weighted MRI sequences. T2/FLAIR enhancing abnormalities (excluding the contrast-enhancing area as well as the necrotic area of the tumor mass) were calculated on T2-/FLAIR-weighted images. In case of more than one lesion, each volume was summed up in order to calculate one total tumor volume [23]. Measurable disease was defined as a tumor volume ≥1 cm^3^ [30]. Tumor segmentation was performed using a semiautomated active contour method (ITK-SNAP 3.8.0), which demonstrated excellent reliability and high efficiency of 3D segmentation [31].

### 2.5. Statistical Analysis

Results of OS, PFS and postprogression survival (PPS) are reported as median with 95% confidence intervals (CIs). Distribution of OS, PFS and PPS were calculated by the Kolmogorov–Smirnov test. We calculated PPS, the survival time after progression (time between date of tumor progression and date of death/loss to follow-up) in order to evaluate which assessment criteria is superior in predicting OS. We assumed, if PPS is short, the time point of progression assessed by MRI is a reliable surrogate endpoint. Differences in PFS and PPS among the assessment criteria were calculated by the Kruskal–Wallis test and results were corrected for multiple comparison (Bonferroni’s adjustment). For correlation analysis between PFS and OS, the Spearman test was used. Median PFS and PPS was calculated for all patients included in the trial (*n* = 76) and for the Audencel subgroup, comprised only of patients included in the SOC + Audencel arm (*n* = 36).

Landmark analysis was carried out to detect whether progression within a 4- or 8-months interval has an influence on survival. Patients who had died prior to these specific landmarks were excluded from analysis. At the landmark time of 4 and 8 months, response (PD or SD) according to each assessment criteria was computed for each patient and residual survival time was calculated (date of 4- or 8-months after pre-surgery MRI to date of death/loss to follow-up/end of study).

To evaluate whether there were differences between the response criteria in predicting OS, Cox proportional hazard models were used to calculate hazard ratios (HRs) and their corresponding 95% CIs. The Audencel subgroup was too small for further statistical analysis. Statistical analysis was performed using SPSS Statistics v27.0 (IBM).

## 3. Results

### 3.1. Patient Characteristics

In total, 105 patients were enrolled in the Audencel trial. The required inclusion criteria for this analysis were met for 76 (27 female, 49 male) patients, while 40 patients were treated with SOC, 36 patients received SOC + Audencel vaccine. All patients had histologically confirmed primary GB. In Table 2, clinical and demographic information of patients included in the Audencel trial is displayed. Patients had undergone a median of eight follow-up MRI scans (range 2–15), with a median time interval of 2.7 months [2.4–2.8] between each MRI scan.

### 3.2. Progression-Free Survival and Postprogression Survival

All patients had undergone gross total tumor resection. No measurable tumor mass was detected on postsurgery MRI, so the best possible response for every patient was SD.

PFS differed significantly between the individual response-assessment criteria. Overall, there was a significant difference in median PFS between mRANO (8.6 months) and Vol-mRANO (8.6 months) compared to MacDonald (4.0 months), RANO (4.2 months) and Vol-RANO (5.4 months). In the Audencel subgroup, there was a significant difference in median PFS between mRANO (8.1 months) and Vol-mRANO (8.6 months) compared to MacDonald (4.2 months). In Table 3, the specific *p*-values and median PFS with CI for all assessment criteria are listed. Interestingly, there was no difference in PFS between SOC and SOC + Audencel using the different response-assessment criteria.

The difference in PPS between the response-assessment criteria was also statistically different. In the entire cohort, there was a significant difference in median PPS between mRANO (8.8 months) and Vol-mRANO (8.7 months) compared to MacDonald (12.0 months), RANO (11.4 months) and Vol-RANO (10.8 months). In the Audencel subgroup, there was a significant difference in median PPS between Vol-mRANO (6.2 months) and mRANO (7.3 months) compared to MacDonald (15.2 months). Median PPS by Vol-mRANO (6.2 months) was also significantly shorter compared to RANO (12.3 months), Vol-RANO (12.1 months) and iRANO (13.0 months). In Table 4, the specific *p*-values and median PPS with CI for all assessment criteria are listed.

### 3.3. Progression-Free Survival and Correlation with Overall survival

The best correlation between PFS and OS was detected for Vol-mRANO (r = 0.69) and mRANO (r = 0.65, Spearman test, *p* < 0.0001) followed by MacDonald (r = 0.44), RANO (r = 0.45), Vol-RANO (r = 0.46) and iRANO (r = 0.50, Spearman test, *p* < 0.0001).

### 3.4. Landmark Analysis

Response status (SD or PD) was determined for each patient at the 4- and 8-month landmark time. In total, at the 4-month landmark 75 (98.7%) patients and at the 8-month landmark 71 (93.4%) patients were included. For iRANO (*n* = 36), at 4 months, 35 (97.2%) patients were included; and at 8 months, 32 (88.9%) patients were included.

By using Cox proportional hazard models, a correlation between progression status (PD or SD) at the specific landmark time and OS was detected. HR, *p*-values and their corresponding 95% CIs for the 4- and 8-month landmark time are summarized in Table 5. The highest HR for PD was observed for mRANO (HR = 2.57, *p* < 0.001) and Vol-mRANO (HR = 2.79, *p* < 0.001) at the 8-month landmark time; however, the difference between each HR for all response-assessment criteria was not significant (*p* = 0.46).

The impact of SD or PD on median OS at the 4- and 8-month landmark was calculated and listed in Table 6. There was no significant difference between median OS, for patients with PD or SD, assessed by different response-assessment criteria. However, at the 4-month landmark time the impact of progressive disease on median OS was most distinct for mRANO, Vol-mRANO and iRANO, and at the 8-month landmark time for mRANO and Vol-mRANO. For those criteria, the greatest difference in OS between SD and PD at the specific landmark time was observed.

### 3.5. Non-Enhancing Abnormalities

In 16 patients (21.1%) volumetric T2/FLAIR changes (Vol-RANO), and in 13 patients (17.1%) a significant increase in T2/FLAIR changes (RANO), were seen prior to detection of a contrast-enhancing lesion on postgadolinium T1-weighted MRI scans. In those patients, T2/FLAIR changes appeared for Vol-RANO 10.5 months (median, range 1.4–39.3 months) and for RANO 9.8 months (median, range 2.0–32.6 months) prior to the T1 contrast-enhancing lesion. Moreover, 11/16 (Vol-RANO) and 8/13 (RANO) patients showed a disease progression on postgadolinium T1-weighted MRI scans later in the disease course.

**Figure 2:**
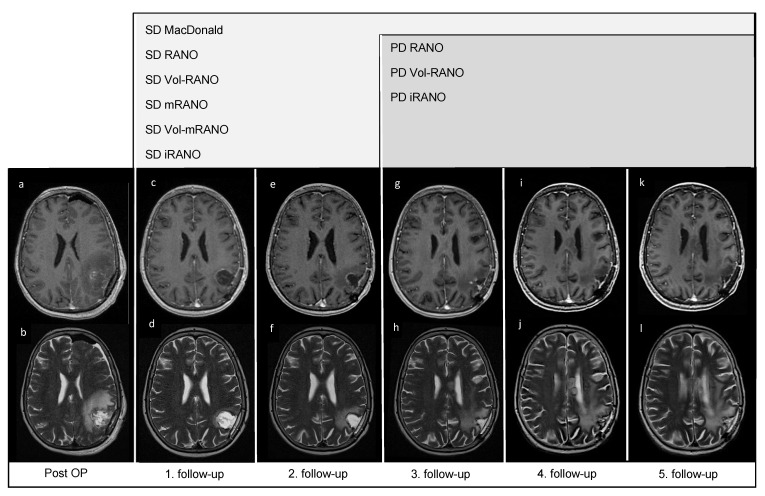
Post-OP and follow-up MRI scans of patient VAX_0066 (Audencel-arm): T2- (**b**,**d**,**f**,**h**,**j**,**l**) and postgadolinium T1-weighted MRI sequences (**a**,**c**,**e**,**g**,**i**,**k**) are displayed. This figure illustrates the progression of non-enhancing abnormalities. At the first follow-up MRI (**c**,**d**) non-enhancing abnormalities are decreased and no contrast-enhancing tumor mass is seen compared to post-OP (**a**,**b**) where no measurable disease is seen. Hence, the patient is defined as stable disease (SD) by all assessment criteria.Therefore, the first follow-up MRI (**c**,**d**) is used as baseline MRI, as it shows the best response. The second follow-up MRI (**e**,**f**) still shows SD compared to baseline (**c**,**d**). At the third follow-up MRI (**g**,**h**) an increase in non-enhancing abnormalities (corpus callosum, (**h**)) compared to T2-weighted sequence of the first follow-up (**d**) is seen. On the fourth- (**i**,**j**) and fifth follow-up scans (**k**,**l**), T2-changes are further increased (**j**,**l**). On T1-weighted MRI scans from first to fifth follow-up (**c**,**e**,**g**,**i**,**k**), no measurable contrast-enhancing tumor mass is seen, including the last T1-weighted follow-up MRI scan (**k**).

In Figure 2, five follow-up MRI scans of a representative patient (VAX_0066, Audencel-arm) are displayed. In this patient, tumor progression was observed only as a non-enhancing abnormality, thus the addition of T2-weighted sequences was beneficial in this case.

### 3.6. Pseudoprogression

By applying mRANO and Vol-mRANO criteria 19 (25.0%) and 23 (30.3%) patients had confirmed PsP, respectively. When iRANO was applied to patients treated with SOC + Audencel, 4 (11.1%) patients had confirmed PsP. The median OS for patients with confirmed PsP by mRANO was 23.4 months (95% CI, 19.0–31.1), for Vol-mRANO 21.2 months (95% CI, 18.1–28.7), and for patients without PsP 17.9 months (95% CI, 16.2–22.8). No significant difference in median OS between patients with confirmed PsP (mRANO and Vol-mRANO) and patients without PsP was seen.

## 4. Discussion

Over the last decades, several response-assessment criteria have been proposed, in order to more precisely define the time point of disease progression in malignant glioma [20,21,23,25]. In this post hoc analysis, we evaluated the different response-assessment criteria and their ability to predict OS in patients with newly diagnosed glioblastoma treated with SOC and SOC + tumor lysate-charged autologous dendritic cells in a multicenter phase II clinical trial. The best correlation between PFS and OS was observed for mRANO and Vol-mRANO. No difference in PFS between SOC and SOC + Audencel was seen using the different response criteria. Interestingly, iRANO was not superior in predicting PFS for patients in the Audencel arm.

Reliable study endpoints are crucial in order to evaluate tumor progression and determine the effectiveness of new treatments. Although the main goal of new cancer treatment approaches is an improvement in OS, the estimation of surrogate endpoints might also show some advantages [32,33,34]. For instance, PFS plays an important role in neuro-oncologic trials, as it can be assessed earlier and is independent of subsequent post-progression treatment [34]. Consequently, response-assessment methods that evaluate PFS must be reliable, as therapy should only be changed or discontinued if progression is confirmed. In most cases, MRI provides a valuable first approach to evaluate the tumor-response status. In selected cases, however, O-(2-[18F] fluoroethyl)-L-tyrosine-positron emission tomography (18F-FET-PET) imaging or even tumor biopsy are used to confirm or exclude tumor progression [35].

Several studies already compared various response-assessment criteria in neuro-oncology with different results. In a cohort of 102 patients with recurrent GB treated with a combination of bevacizumab and irinotecan, 1-dimensional and 2-dimensional criteria (Macdonald, RECIST, RANO and RECIST + F criteria) were compared [36]. In this retrospective analysis, no difference in median PFS between those criteria could be demonstrated. Radiologic data of 163 patients with recurrent GB treated with irinotecan combined with bevacizumab from the randomized phase II BRAIN (AVF3708g) trial were evaluated for pure T2/FLAIR progression by comparing RANO and MacDonald criteria [28]. It was shown that the median PFS assessed by RANO was shorter compared to the median PFS assessed by MacDonald criteria. A total of 35% of those patients only progressed as defined by RANO and had a T2/FLAIR progression. Recently, the response assessment criteria mRANO, iRANO and RANO were compared by Ellingson et al. [29] in 47 patients with recurrent GB, who were treated with the IL4R-targeted immunotoxin MDNA55. Again, median PFS by RANO was significantly shorter compared to median PFS assessed by mRANO or iRANO. Additionally, a correlation between OS and mRANO PFS was detected.

We could reproduce these findings by showing that median PFS was shorter for MacDonald, RANO and Vol-RANO compared to mRANO and Vol-mRANO in a cohort of 76 patients. Furthermore, we also detected a strong correlation between OS and PFS assessed by Vol-mRANO and mRANO. In our landmark analysis, the HR for patients with a confirmed PD was highest when assessed by mRANO and Vol-mRANO, and the impact of PD at a specific landmark time on OS was best shown by mRANO and Vol-mRANO. Interestingly, in the subgroup of Audencel-treated patients, iRANO was not superior in predicting OS. Our study results could indicate that mRANO and Vol-mRANO may represent the most valid response-assessment criteria in GB patients treated with SOC or additional dendritic-cell-vaccine therapy. However, in order to draw more reliable conclusions on the value of iRANO, larger patient cohorts treated by different immunotherapy approaches need to be investigated.

The difference in PFS can in part be explained by the definition of the criteria, as mRANO and iRANO require a confirmatory scan after the first appearance of progression or regression of the enhancing tumor mass [23]. iRANO criteria also require the confirmation of progression by an additional MRI scan at least 3 months after the first appearance of a new enhancing lesion or the increase in size of a tumor lesion [25].

The differentiation of PsP from true progression can further challenge the treatment of patients with GB [37], and in particular, patients treated with immunotherapy can show unique patterns of PsP [24,25]. In our analysis, 25.0% and 30.3% (mRANO and Vol-mRANO, respectively) of our patients showed signs of PsP. Patients who received the dendritic-cell vaccine showed PsP by iRANO in only 11.1%. OS was identical for patients with PsP compared to those patients without PsP. This is in line with results from the cohort of Ellingson et al. [29] where 45.2% (mRANO) and 9.5% (iRANO) of patients treated with an IL4R-targeted immunotoxin had confirmed PsP.

The challenge to interpret the appearance of pure non-enhancing abnormalities before or even without development of a contrast-enhancing tumor mass was first recognized during bevacizumab therapy [38,39]. By using RANO assessment criteria we observed that 21.1% of our patients had T2/FLAIR changes prior to or without progression on the postgadolinium T1-weighted MRI sequences, which is comparable to other studies [28,40]. As most of the patients included in the Audencel trial did also receive bevacizumab therapy during disease progression, it is difficult to determine whether antiangiogenic therapy or multifactorial effects are responsible for these findings.

Because GB frequently shows an inhomogeneous and complex growth pattern, several studies already evaluated whether volumetric-measurement approaches show better performance in assessing progression compared to 1D (the overall largest diameter measured on axial slices) and/or 2D measurement approaches [41,42,43,44,45]. Gahrmann et al. [42] compared 2D RANO criteria to four different volumetric approaches based on enhancement, subtraction and T2/FLAIR abnormalities, but they were not able to demonstrate an improvement in predicting OS by using those volumetric approaches. Similarly, in our study, there was no difference in median PFS between RANO and Vol-RANO, as well as between mRANO and Vol-mRANO. Although semiautomatic volumetric-measurement techniques are available nowadays for more precise tumor-volume assessment, this seems to be of limited clinical utility in this context. In addition, these techniques are also time-consuming and more complex than 2D measurement approaches. Moreover, tumor volumes can vary between different MRI scans because of different angulations and slice thicknesses. Recently, artificial intelligence (AI) advanced the field of volumetric measurements. Kickingereder et al. [30] and Chang et al. [46] investigated methods to automatically assess tumor response using deep-learning approaches and artificial neural networks with promising results. Tumor volumes measured by AI were superior in predicting OS compared to radiologic-response assessment by RANO performed by a radiologist [30]. Despite great advances reported with AI, caution has to be taken in generalizing these models, due to lack of standardization for data acquisition, feature extraction and analysis methods used [47,48,49].

There are some limitations to consider in this post hoc analysis. In general, it is a retrospective evaluation of radiologic data generated in a national multicenter study. Only radiologic data was used for response assessment, as information about clinical appearance and steroid dosage was barely available. Hence, the purpose of our post hoc analysis is a validation of imaging parameters for response assessment in neuro-oncology, while neurologic function and patient quality of life have been validated in other studies (e.g., Ung et al. [50]). Imaging acquisition was not standardized, and only in some patients were MP-RAGE MRI sequences available, while for others only T1-weighted SE images were carried out. In this trial, MacDonald criteria were initially used for response assessment, and MRI scans were not carried out in close proximity to the end of radiation therapy. Consequently, we used postsurgery MRI as baseline for all response-assessment criteria in order to facilitate a better comparison between the different criteria. Furthermore, only half of the patients included in this trial were treated with an autologous dendritic-cell vaccine. Therefore, we were only able to apply iRANO on a small number of patients. To allow a more reliable conclusion, whether iRANO is better for response assessment compared to other assessment methods in the context of immunotherapy, larger patient cohorts and inclusion of different immunotherapy treatment strategies will be needed.

## 5. Conclusions

We conclude that mRANO criteria are superior to MacDonald and RANO for predicting progression in patients with newly diagnosed GB treated with SOC ± additional Audencel-based immunotherapy. Moreover, the best correlation between PFS and OS was seen for mRANO and Vol-mRANO. Between the two treatment arms, no difference in PFS and OS was seen and iRANO was not superior for predicting OS in patients treated with Audencel. These findings, however, need to be confirmed in a larger patient cohort including different immunotherapy approaches.

## Figures and Tables

**Table 1:** Summary of response-assessment criteria.

Response Criteria	Complete Response	Partial Response	Stable Disease	Progressive Disease
MacDonald [20]	disappearance of all enhancing tumor	≥50% decrease in cross-section area of measurable disease	not qualified for other	≥25% increase in cross-section area; new lesion
Vol-RANO [30], RANO [21]	disappearance of measurable and nonmeasurable disease; no new lesion; stable/improved non-enhancing T2/FLAIR abnormalities	≥50% decrease in cross-section area of measurable disease; no progress of nonmeasurable disease; stable/improved T2/FLAIR abnormalities	not qualified for other; stable T2/FLAIR abnormalities; best response for patients with nonmeasurable disease at baseline	≥25% increase in cross-section area/≥40% increase in total volume; new lesion; significant increase or ≥100% increase in volume of T2/FLAIR abnormalities
Vol-mRANO, mRANO [23]	1. MRI: Preliminary CR disappearance of all measurable and nonmeasurable disease 2. MRI (4–8 weeks later): if continuous disappearance: durable CR; if measurable disease: preliminary PD/ pseudoresponse	1. MRI Preliminary PR. ≥50% decrease in cross-section area/≥65% decrease in total volume of measurable disease 2. MRI (4–8 weeks later): if SD, PR or CR: durable PR; if PD: preliminary PD/ pseudoresponse	not qualified for other; best response for patients with nonmeasurable disease at baseline	1. MRI: Preliminary PD new measurable lesion; ≥25% increase in cross-section area/≥40% increase in total volume 2. MRI (4–8 weeks later): if subsequent ≥25% in cross-section area/≥40% increase in total volume: confirmed PD; if SD or PR/CR: pseudoprogression
iRANO [25]	disappearance of measurable and nonmeasurable disease; no new lesion; stable/improved non-enhancing T2/FLAIR abnormalities	≥50% decrease in cross-section area of measurable disease; no progress of nonmeasurable disease; stable/improved T2/FLAIR abnormalities	not qualified for other; stable T2/FLAIR abnormalities; best response for patients with nonmeasurable disease at baseline	1. MRI within 6 months of treatment start: ≥25% increase in cross-section area; new lesion; significant increase in non-enhancing T2/FLAIR abnormalities additional 2. MRI in ≥3 months: if RANO for PD met: PD; if RANO for SD, PD, CR met: pseudoprogression MRI after 6 months of treatment start: ≥25% increase in cross-section area; new lesion; significant increase in non-enhancing T2/FLAIR abnormalities

RANO: Response Assessment in Neuro-Oncology, mRANO: modified Response Assessment in Neuro-Oncology, iRANO: Immunotherapy Response Assessment in Neuro-Oncology, CR: complete response, PR: partial response, SD: stable disease, PD: progressive disease.

**Table 2:** Clinical and demographic information of the study population.

Characteristics		Audencel Group	Control Group
Number of patients		36	40
Sex, *n* (%)	male	20 (55.6)	29 (72.5)
	female	16 (44.4)	11 (27.5)
Median age at diagnosis, years (95% CI)		59.4 (53.6–61.5)	54.4 (50.5–57.0)
Median overall survival, months (95% CI)		18.7 (17.7–27.0)	19.3 (16.5–23.4)
Survival at trial end, *n* (%)	death	30 (83.3)	31 (77.5)
	alive	4 (11.1)	6 (15)
	unknown	2 (5.6)	3 (7.5)
ECOG at baseline, *n* (%)	0	11 (30.6)	15 (37.5)
	1	25 (69.4)	20 (50)
	2	0 (0)	5 (12.5)
MGMT promoter, *n* (%)	samples measured	20	17
	methylated	7/20 (35)	6/17 (35.3)
	unmethylated	13/20 (65)	11/17 (64.7)
IDH 1 mutation, *n* (%)	yes	0 (0)	0 (0)
	no	36 (100)	40 (100)
Side of tumor bulk, *n* (%)	left	16 (44.4)	22 (55)
	right	18 (50)	18 (45)
	central/bilateral	2 (5.6)	0 (0)
Tumor location, *n* (%)	frontal	10 (27.8)	17 (42.5)
	temporal	4 (11.1)	5 (12.5)
	parietal	8 (22.2)	8 (20)
	occipital	14 (38.9)	10 (25)

ECOG: Eastern Cooperative Oncology Group, MGMT: O-6-methylguanine-DNA methyltransferase, IDH 1: isocitrate dehydrogenase 1, *n*: number of patients.

**Table 3:** Median progression-free survival with the corresponding confidence interval for the different assessment criteria. Calculated *p*-values (Kruskal–Wallis test) and corrected for multiple testing (Bonferroni’s adjustment) for difference in PFS between assessment criteria.

Response Criteria	Median PFS, Months	95% CI	Difference of PFS (*p*-Value)					
			MacDonald	RANO	Vol-RANO	mRANO	Vol-mRANO	iRANO
**SOC and SOC + Audencel Patients (*n* = 76)**								
MacDonald	4.0	5.2–8.8	-	1.000	1.000	**0.001**	**0.000**	-
RANO	4.2	5.3–8.6	1.000	-	1.000	**0.003**	**0.001**	-
Vol-RANO	5.4	5.4–8.2	1.000	1.000	-	**0.022**	**0.008**	-
mRANO	8.6	9.1–14.0	**0.001**	**0.003**	**0.022**	-	1.000	-
Vol-mRANO	8.6	9.7–14.9	**0.000**	1.000	**0.008**	1.000	-	-
**SOC + Audencel patients (*n* = 36)**								
MacDonald	4.2	4.2–10.3	-	1.000	1.000	**0.034**	**0.020**	1.000
RANO	4.7	4.6–10.6	1.000	-	1.000	0.105	0.066	1.000
Vol-RANO	5.4	4.5–9.0	1.000	1.000	-	0.154	0.095	1.000
mRANO	8.1	8.6–17.8	**0.034**	0.105	0.154	-	1.000	1.000
Vol-mRANO	8.6	9.4–19.1	**0.020**	0.066	0.154	1.000	-	1.000
iRANO	6.2	5.7–11.7	1.000	1.000	1.000	1.000	1.000	-

PFS: progression-free survival, CI: confidence interval, SOC: standard of care, *n*: number of patients. Significant *p*-values are marked with bold characters.

**Table 4:** Median postprogression survival with the corresponding confidence interval for the different assessment criteria. Calculated *p*-values (Kruskal–Wallis test) and corrected for multiple testing (Bonferroni’s adjustment) for difference in PPS between assessment criteria.

Response Criteria	Median PPS, Months	95% CI	Difference of PPS (*p*-Value)					
			MacDonald	RANO	Vol-RANO	mRANO	Vol-mRANO	iRANO
**SOC and SOC + Audencel Patients (*n* = 76)**								
MacDonald	12.0	11.8–15.8	-	1.000	1.000	**0.013**	**0.001**	-
RANO	11.4	11.8–15.9	1.000	-	1.000	**0.019**	**0.002**	-
Vol-RANO	10.8	11.7–16.2	1.000	1.000	-	**0.046**	**0.005**	-
mRANO	8.8	7.8–11.2	**0.013**	**0.019**	**0.046**	-	1.000	-
Vol-mRANO	8.7	7.1–10.4	**0.001**	**0.002**	**0.005**	1.000	-	-
**SOC + Audencel patients (*n* = 36)**								
MacDonald	15.2	11.9–17.2	-	1.000	1.000	**0.030**	**0.002**	1.000
RANO	12.3	11.4–17.0	1.000	-	1.000	0.104	**0.011**	1.000
Vol-RANO	12.1	11.4–18.8	1.000	1.000	-	0.137	**0.015**	1.000
mRANO	7.3	6.6–11.6	**0.030**	0.104	0.137	-	**1.000**	0.351
Vol-mRANO	6.2	5.6–10.5	**0.002**	**0.011**	**0.015**	1.000	**-**	0.048
iRANO	13.0	10.6–16.2	1.000	1.000	1.000	0.351	**0.048**	-

PPS: postprogression survival, CI: confidence interval, SOC: standard of care, *n*: Number of patients. Significant *p*-values are marked with bold characters.

**Table 5:** Hazard ratios with corresponding confidence interval for patients with progressive disease at the 4- and 8-month landmark time.

Response Criteria	4-Month Landmark			8-Month Landmark		
	HR	95% CI	*p*-Value	HR	95% CI	*p*-Value
MacDonald	1.30	0.79–2.13	0.310	2.29	1.34–3.91	**0.002**
RANO	1.41	0.86–2.33	0.175	2.04	1.18–3.55	**0.011**
Vol-RANO	1.30	0.78–2.15	0.312	1.81	1.06–3.10	**0.031**
mRANO	1.69	0.96–2.96	0.068	2.57	1.48–4.46	**0.001**
Vol-mRANO	1.82	1.01–3.27	**0.045**	2.79	1.59–4.89	**0.001**
iRANO	2.07	0.98–4.37	0.057	1.20	0.88–4.53	0.098

HR: hazard ratio, CI: confidence interval. Significant *p*-values are marked with bold characters.

**Table 6:** Impact of stable disease or progressive disease on median overall survival at 4- and 8-month landmark time with corresponding confidence interval.

Response Criteria	Median OS, Months (95% CI)			
	4-Month Landmark		8-Month Landmark	
	SD	PD	SD	PD
MacDonald	20.5 (18.5–26.9)	18.6 (15.8–22.8)	23.7 (21.4–30.7)	18.0 (15.5–20.9)
RANO	21.5 (19.6–27.7)	15.0 (14.8–21.8)	24.1 (22.5–33.7)	18.1 (15.9–21.0)
Vol-RANO	20.7 (19.3–27.1)	15.0 (14.6–21.8)	23.5 (21.8–31.4)	17.9 (16.1–22.4)
mRANO	20.4 (19.0–25.4)	13.6 (12.5–22.0)	22.8 (21.4–28.6)	13.7 (13.1–19.0)
Vol-mRANO	20.6 (19.1–25.4)	12.8 (11.2–21.5)	23.1 (22.1–29.3)	12.0 (12.5–17.9)
iRANO	21.7 (19.1–31.0)	12.7 (11.0–20.9)	23.4 (19.2–40.5)	17.3 (15.0–22.7)

SD: stable disease, PD: progressive disease, OS: overall survival.

## Data Availability

The datasets analyzed during the current study are available from the corresponding author on reasonable request.

## References

[1] Wöhrer A., Waldhör T., Heinzl H., Hackl M., Feichtinger J., Gruber-Mösenbacher U., Kiefer A., Maier H., Motz R., Reiner-Concin A. (2009). The Austrian Brain Tumour Registry: A cooperative way to establish a population-based brain tumour registry. J. Neurooncol..

[2] Ostrom Q.T., Patil N., Cioffi G., Waite K., Kruchko C., Barnholtz-Sloan J.S. (2020). CBTRUS Statistical Report: Primary Brain and Other Central Nervous System Tumors Diagnosed in the United States in 2013–2017. Neuro Oncol..

[3] Stupp R., Mason W.P., van den Bent M.J., Weller M., Fisher B., Taphoorn M.J., Belanger K., Brandes A.A., Marosi C., Bogdahn U. (2005). Radiotherapy plus concomitant and adjuvant temozolomide for glioblastoma. N. Engl. J. Med..

[4] Stupp R., Taillibert S., Kanner A.A., Kesari S., Steinberg D.M., Toms S.A., Taylor L.P., Lieberman F., Silvani A., Fink K.L. (2015). Maintenance Therapy With Tumor-Treating Fields Plus Temozolomide vs Temozolomide Alone for Glioblastoma: A Randomized Clinical Trial. JAMA.

[5] Herrlinger U., Tzaridis T., Mack F., Steinbach J.P., Schlegel U., Sabel M., Hau P., Kortmann R.D., Krex D., Grauer O. (2019). Lomustine-temozolomide combination therapy versus standard temozolomide therapy in patients with newly diagnosed glioblastoma with methylated MGMT promoter (CeTeG/NOA-09): A randomised, open-label, phase 3 trial. Lancet.

[6] Wen P.Y., Weller M., Lee E.Q., Alexander B.M., Barnholtz-Sloan J.S., Barthel F.P., Batchelor T.T., Bindra R.S., Chang S.M., Chiocca E.A. (2020). Glioblastoma in adults: A Society for Neuro-Oncology (SNO) and European Society of Neuro-Oncology (EANO) consensus review on current management and future directions. Neuro Oncol..

[7] Liau L.M., Ashkan K., Tran D.D., Campian J.L., Trusheim J.E., Cobbs C.S., Heth J.A., Salacz M., Taylor S., D’Andre S.D. (2018). First results on survival from a large Phase 3 clinical trial of an autologous dendritic cell vaccine in newly diagnosed glioblastoma. J. Transl. Med..

[8] Wen P.Y., Reardon D.A., Armstrong T.S., Phuphanich S., Aiken R.D., Landolfi J.C., Curry W.T., Zhu J.J., Glantz M., Peereboom D.M. (2019). A Randomized Double-Blind Placebo-Controlled Phase II Trial of Dendritic Cell Vaccine ICT-107 in Newly Diagnosed Patients with Glioblastoma. Clin. Cancer Res..

[9] Akasaki Y., Kikuchi T., Homma S., Koido S., Ohkusa T., Tasaki T., Hayashi K., Komita H., Watanabe N., Suzuki Y. (2016). Phase I/II trial of combination of temozolomide chemotherapy and immunotherapy with fusions of dendritic and glioma cells in patients with glioblastoma. Cancer Immunol. Immunother..

[10] Ardon H., Van Gool S.W., Verschuere T., Maes W., Fieuws S., Sciot R., Wilms G., Demaerel P., Goffin J., Van Calenbergh F. (2012). Integration of autologous dendritic cell-based immunotherapy in the standard of care treatment for patients with newly diagnosed glioblastoma: Results of the HGG-2006 phase I/II trial. Cancer Immunol. Immunother..

[11] Cao J.X., Zhang X.Y., Liu J.L., Li D., Li J.L., Liu Y.S., Wang M., Xu B.L., Wang H.B., Wang Z.X. (2014). Clinical efficacy of tumor antigen-pulsed DC treatment for high-grade glioma patients: Evidence from a meta-analysis. PLoS ONE.

[12] Inogés S., Tejada S., de Cerio A.L., Gállego Pérez-Larraya J., Espinós J., Idoate M.A., Domínguez P.D., de Eulate R.G., Aristu J., Bendandi M. (2017). A phase II trial of autologous dendritic cell vaccination and radiochemotherapy following fluorescence-guided surgery in newly diagnosed glioblastoma patients. J. Transl. Med..

[13] Rapp M., Grauer O.M., Kamp M., Sevens N., Zotz N., Sabel M., Sorg R.V. (2018). A randomized controlled phase II trial of vaccination with lysate-loaded, mature dendritic cells integrated into standard radiochemotherapy of newly diagnosed glioblastoma (GlioVax): Study protocol for a randomized controlled trial. Trials.

[14] Buchroithner J., Erhart F., Pichler J., Widhalm G., Preusser M., Stockhammer G., Nowosielski M., Iglseder S., Freyschlag C.F., Oberndorfer S. (2018). Audencel Immunotherapy Based on Dendritic Cells Has No Effect on Overall and Progression-Free Survival in Newly Diagnosed Glioblastoma: A Phase II Randomized Trial. Cancers.

[15] Hygino da Cruz L.C., Rodriguez I., Domingues R.C., Gasparetto E.L., Sorensen A.G. (2011). Pseudoprogression and pseudoresponse: Imaging challenges in the assessment of posttreatment glioma. AJNR Am. J. Neuroradiol..

[16] Wick W., Chinot O.L., Bendszus M., Mason W., Henriksson R., Saran F., Nishikawa R., Revil C., Kerloeguen Y., Cloughesy T. (2016). Evaluation of pseudoprogression rates and tumor progression patterns in a phase III trial of bevacizumab plus radiotherapy/temozolomide for newly diagnosed glioblastoma. Neuro Oncol..

[17] Sanghera P., Perry J., Sahgal A., Symons S., Aviv R., Morrison M., Lam K., Davey P., Tsao M.N. (2010). Pseudoprogression following chemoradiotherapy for glioblastoma multiforme. Can. J. Neurol. Sci..

[18] Ellingson B.M., Chung C., Pope W.B., Boxerman J.L., Kaufmann T.J. (2017). Pseudoprogression, radionecrosis, inflammation or true tumor progression? challenges associated with glioblastoma response assessment in an evolving therapeutic landscape. J. Neurooncol..

[19] Nowosielski M., Wen P.Y. (2018). Imaging Criteria in Neuro-oncology. Semin. Neurol..

[20] Macdonald D.R., Cascino T.L., Schold S.C., Cairncross J.G. (1990). Response criteria for phase II studies of supratentorial malignant glioma. J. Clin. Oncol..

[21] Wen P.Y., Macdonald D.R., Reardon D.A., Cloughesy T.F., Sorensen A.G., Galanis E., Degroot J., Wick W., Gilbert M.R., Lassman A.B. (2010). Updated response assessment criteria for high-grade gliomas: Response assessment in neuro-oncology working group. J. Clin. Oncol..

[22] Clarke J.L., Chang S. (2009). Pseudoprogression and pseudoresponse: Challenges in brain tumor imaging. Curr. Neurol. Neurosci. Rep..

[23] Ellingson B.M., Wen P.Y., Cloughesy T.F. (2017). Modified Criteria for Radiographic Response Assessment in Glioblastoma Clinical Trials. Neurotherapeutics.

[24] Wolchok J.D., Hoos A., O’Day S., Weber J.S., Hamid O., Lebbé C., Maio M., Binder M., Bohnsack O., Nichol G. (2009). Guidelines for the evaluation of immune therapy activity in solid tumors: Immune-related response criteria. Clin. Cancer Res..

[25] Okada H., Weller M., Huang R., Finocchiaro G., Gilbert M.R., Wick W., Ellingson B.M., Hashimoto N., Pollack I.F., Brandes A.A. (2015). Immunotherapy response assessment in neuro-oncology: A report of the RANO working group. Lancet Oncol..

[26] Thomas R., Somarouthu B., Alessandrino F., Kurra V., Shinagare A.B. (2019). Atypical Response Patterns in Patients Treated With Nivolumab. AJR Am. J. Roentgenol..

[27] Ma Y., Wang Q., Dong Q., Zhan L., Zhang J. (2019). How to differentiate pseudoprogression from true progression in cancer patients treated with immunotherapy. Am. J. Cancer Res..

[28] Huang R.Y., Rahman R., Ballman K.V., Felten S.J., Anderson S.K., Ellingson B.M., Nayak L., Lee E.Q., Abrey L.E., Galanis E. (2016). The Impact of T2/FLAIR Evaluation per RANO Criteria on Response Assessment of Recurrent Glioblastoma Patients Treated with Bevacizumab. Clin. Cancer Res..

[29] Ellingson B.M., Sampson J., Achrol A.S., Aghi M.K., Bankiewicz K., Wang C., Bexon M., Brem S., Brenner A., Chowdhary S. (2021). Modified RANO, Immunotherapy RANO, and Standard RANO Response to Convection-Enhanced Delivery of IL4R-Targeted Immunotoxin MDNA55 in Recurrent Glioblastoma. Clin. Cancer Res..

[30] Kickingereder P., Isensee F., Tursunova I., Petersen J., Neuberger U., Bonekamp D., Brugnara G., Schell M., Kessler T., Foltyn M. (2019). Automated quantitative tumour response assessment of MRI in neuro-oncology with artificial neural networks: A multicentre, retrospective study. Lancet Oncol..

[31] Yushkevich P.A., Piven J., Hazlett H.C., Smith R.G., Ho S., Gee J.C., Gerig G. (2006). User-guided 3D active contour segmentation of anatomical structures: Significantly improved efficiency and reliability. Neuroimage.

[32] Prados M., Cloughesy T., Samant M., Fang L., Wen P.Y., Mikkelsen T., Schiff D., Abrey L.E., Yung W.K., Paleologos N. (2011). Response as a predictor of survival in patients with recurrent glioblastoma treated with bevacizumab. Neuro Oncol..

[33] Lamborn K.R., Yung W.K., Chang S.M., Wen P.Y., Cloughesy T.F., DeAngelis L.M., Robins H.I., Lieberman F.S., Fine H.A., Fink K.L. (2008). Progression-free survival: An important end point in evaluating therapy for recurrent high-grade gliomas. Neuro Oncol..

[34] Han K., Ren M., Wick W., Abrey L., Das A., Jin J., Reardon D.A. (2014). Progression-free survival as a surrogate endpoint for overall survival in glioblastoma: A literature-based meta-analysis from 91 trials. Neuro Oncol..

[35] Weller M., van den Bent M., Preusser M., Le Rhun E., Tonn J.C., Minniti G., Bendszus M., Balana C., Chinot O., Dirven L. (2021). EANO guidelines on the diagnosis and treatment of diffuse gliomas of adulthood. Nat. Rev. Clin. Oncol..

[36] Gállego Pérez-Larraya J., Lahutte M., Petrirena G., Reyes-Botero G., González-Aguilar A., Houillier C., Guillevin R., Sanson M., Hoang-Xuan K., Delattre J.Y. (2012). Response assessment in recurrent glioblastoma treated with irinotecan-bevacizumab: Comparative analysis of the Macdonald, RECIST, RANO, and RECIST + F criteria. Neuro Oncol..

[37] Brandsma D., van den Bent M.J. (2009). Pseudoprogression and pseudoresponse in the treatment of gliomas. Curr. Opin. Neurol..

[38] Bergers G., Hanahan D. (2008). Modes of resistance to anti-angiogenic therapy. Nat. Rev. Cancer.

[39] Carmeliet P., Jain R.K. (2011). Molecular mechanisms and clinical applications of angiogenesis. Nature.

[40] Tensaouti F., Khalifa J., Lusque A., Plas B., Lotterie J.A., Berry I., Laprie A., Cohen-Jonathan Moyal E., Lubrano V. (2017). Response Assessment in Neuro-Oncology criteria, contrast enhancement and perfusion MRI for assessing progression in glioblastoma. Neuroradiology.

[41] Dempsey M.F., Condon B.R., Hadley D.M. (2005). Measurement of tumor “size” in recurrent malignant glioma: 1D, 2D, or 3D?. AJNR Am. J. Neuroradiol..

[42] Gahrmann R., van den Bent M., van der Holt B., Vernhout R.M., Taal W., Vos M., de Groot J.C., Beerepoot L.V., Buter J., Flach Z.H. (2017). Comparison of 2D (RANO) and volumetric methods for assessment of recurrent glioblastoma treated with bevacizumab-a report from the BELOB trial. Neuro Oncol..

[43] Boxerman J.L., Zhang Z., Safriel Y., Larvie M., Snyder B.S., Jain R., Chi T.L., Sorensen A.G., Gilbert M.R., Barboriak D.P. (2013). Early post-bevacizumab progression on contrast-enhanced MRI as a prognostic marker for overall survival in recurrent glioblastoma: Results from the ACRIN 6677/RTOG 0625 Central Reader Study. Neuro Oncol..

[44] Galanis E., Buckner J.C., Maurer M.J., Sykora R., Castillo R., Ballman K.V., Erickson B.J. (2006). Validation of neuroradiologic response assessment in gliomas: Measurement by RECIST, two-dimensional, computer-assisted tumor area, and computer-assisted tumor volume methods. Neuro Oncol..

[45] Shah G.D., Kesari S., Xu R., Batchelor T.T., O’Neill A.M., Hochberg F.H., Levy B., Bradshaw J., Wen P.Y. (2006). Comparison of linear and volumetric criteria in assessing tumor response in adult high-grade gliomas. Neuro Oncol..

[46] Chang K., Beers A.L., Bai H.X., Brown J.M., Ly K.I., Li X., Senders J.T., Kavouridis V.K., Boaro A., Su C. (2019). Automatic assessment of glioma burden: A deep learning algorithm for fully automated volumetric and bidimensional measurement. Neuro Oncol..

[47] Despotović I., Goossens B., Philips W. (2015). MRI segmentation of the human brain: Challenges, methods, and applications. Comput. Math. Methods Med..

[48] Li X.T., Huang R.Y. (2020). Standardization of imaging methods for machine learning in neuro-oncology. Neurooncol. Adv..

[49] Klauschen F., Goldman A., Barra V., Meyer-Lindenberg A., Lundervold A. (2009). Evaluation of automated brain MR image segmentation and volumetry methods. Hum. Brain Mapp..

[50] Ung T.H., Ney D.E., Damek D., Rusthoven C.G., Youssef A.S., Lillehei K.O., Ormond D.R. (2019). The Neurologic Assessment in Neuro-Oncology (NANO) Scale as an Assessment Tool for Survival in Patients With Primary Glioblastoma. Neurosurgery.

